# Natural selection and adaptive traits in the Maniq, a nomadic hunter-gatherer society from Mainland Southeast Asia

**DOI:** 10.1038/s41598-024-83657-0

**Published:** 2025-02-09

**Authors:** Tobias Herzog, Maximilian Larena, Wibhu Kutanan, Helmut Lukas, Martin Fieder, Helmut Schaschl

**Affiliations:** 1 Department of Evolutionary Anthropology, Faculty of Life Sciences, University of Vienna, Djerassiplatz 1, Vienna, 1030 Austria; 2 Human Evolution, Department of Organismal Biology, Uppsala University, Norbyvägen 18C, Uppsala, 75236 Sweden; 3 Department of Biology, Faculty of Science, Naresuan University, Phitsanulok, 65000 Thailand; 4 Institute for Social Anthropology, Austrian Academy of Sciences, Georg-Coch-Platz 2, Vienna, 1010 Austria

**Keywords:** Maniq people, Hunter-Gatherer, Genetic adaptation, Positive selection, Balancing selection, Population genetics, Genetic variation, Biological anthropology

## Abstract

Asia is home to diverse hunter-gatherer populations characterized by significant morphological, anthropological, cultural, and linguistic diversity. Despite their importance in understanding ancestral human subsistence, little is known about the essential genetic adaptations of these groups. This study investigates the evolutionary pressures shaping the genome of the Maniq population, a nomadic hunter-gatherer group inhabiting the rainforests of southern Thailand. Using genome-wide approaches, including iHS, xp-EHH, PBE, and beta statistics, we identified signatures of positive and balancing selection. Genes under positive selection were enriched in pathways related to immunity, metabolic regulation, structural adaptation, cardiovascular performance, and neuromodulatory traits. Several genes associated with the Southeast Asian ‘negrito-like’ phenotype were also under positive selection. Balancing selection was primarily detected in immune-related genes, particularly within the HLA region, underscoring the critical role of genetic diversity in surviving pathogen-rich environments. Additionally, balancing selection in olfactory receptor genes highlights their importance in environmental sensing and adaptation. These results reveal the intricate interplay of positive and balancing selection in shaping the genetic landscape of the Maniq population and highlight their adaptations to the ecological and lifestyle challenges of life in the rainforest. This study contributes to our understanding of human evolutionary processes in tropical environments and hunter-gatherer societies.

**Supplementary Information:**

The online version contains supplementary material available at 10.1038/s41598-024-83657-0.

## Introduction

The indigenous people of the Thai-Malay Peninsula in Southeast Asia are known as the ‘Orang Asli’, who are traditionally divided into three groups: the Semang residing in the north, the Senoi in the central region, and the Malay indigenous people, sometimes referred to as ‘Proto-Malay’, residing in the south. The Semang are phenotypically characterized by small body size, dark skin, and tightly curled hair on average, a phenotype which is called ‘negrito’ and also found outside of Malaysia^1^. We recently demonstrated a close genetic relationship between the Maniq, nomadic hunter-gatherers inhabiting the rainforests of southern Thailand, and the Semang^2^. They speak an aboriginal Mon-Khmer language belonging to the Austroasiatic language family. Their society is marked by flexible social groups with regularly changing composition, a lack of reliance on stored food, a system of immediate resource consumption, absence of resource ownership, reciprocal relationships, an egalitarian ethos of sharing, and minimal social hierarchy^3,4^. An estimated 350 individuals of the Maniq society still pursue a nomadic hunter-gatherer lifestyle. Due to the drastic deforestation in Thailand, however, some Maniq have been compelled to alter their settlement patterns, leading to increased sedentarization and departure from their traditional way of life^5^. This transition highlights the importance of understanding their genetic adaptive traits and potential adverse effects associated with transitioning to a modern lifestyle in an industrialized environment.

In this study, we analyze the signatures of natural selection within the Maniq genome, aiming to elucidate their genetic adaptation to the rainforest environment and hunter-gatherer lifestyle. This involved genome-wide searches for signals of both positive directional selection, known as selective sweeps, and balancing selection. Positive selection is the process by which the frequency of a favorable allele increases in a population. This leaves a discernable signal in genomic regions characterized by reduced diversity, longer haplotypes, or pronounced genetic differentiation, often leading to localized genetic adaptation^6^. Previous research has identified genes linked to various traits such as skin pigmentation, nutritional adaptation, olfactory receptors, brain size, and immune function as targets of positive selection in the human genome (see review in^6^). Moreover, the relatively diminutive stature of many rainforest hunter-gatherers is thought to serve as an adaptation to the demanding rainforest habitat. It potentially confers evolutionary advantages by reducing metabolic demands in an environment with limited caloric resources, facilitating thermoregulation, enhancing mobility in dense undergrowth, and aiding in tree climbing^7–11^. One study posited that the small adult body size phenotype across diverse hunter-gatherer populations prompts compensatory changes in cardiac pathways^10^. This suggests convergent adaptations influenced by similar rainforest ecology. Furthermore, signatures of positive selection were found in different African rainforest hunter-gatherers across a set of genes associated with bone synthesis, immunity, reproduction, cell signaling, and energy metabolism^8,12,13^.

In contrast to positive directional selection, which drives the prevalence of specific alleles within a population, balancing selection leads to the maintenance of diversity at a genetic locus in the presence of selection pressure, thereby maintaining multiple variants at intermediate frequencies^14,15^. The mechanisms facilitating this diversity include heterozygote advantage, negative frequency-dependent selection, and spatial or temporal habitat heterogeneity. Notably, genes within the human leukocyte antigen (HLA) system, also known as the major histocompatibility complex (MHC), represent well-documented targets of long-term balancing selection in many vertebrate species including humans. To detect recent signatures of positive directional selection and (long-term) balancing selection in the Maniq genome, we employed the integrated haplotype score (iHS) test^16^, the cross-population extended haplotype homozygosity (xp-EHH) test^17^, the Population Brach Excess (PBE) statistics^18^ and the summary statistic *β*^19,20^, respectively. Our analysis revealed that positive selection was enriched in different biological pathways related to immunity, metabolic regulation, structural integrity, cardiovascular function and neuromodulatory traits, suggesting adaptations to environmental and dietary challenges, pathogen pressure, physical demands, and to the lifestyle as hunter-gatherer. Furthermore, we found signals of balancing selection in genes involved in immune response and immunological signaling pathways. Together with our previous study (see^2^), this study thus provides new insights into the complex evolutionary genetic history and adaptive genetics of the Maniq people, one of the last remaining primary hunter-gatherer societies in contemporary times.

## Results

### Genome-wide signature of positive selection

We identified signatures of positive selection in the Maniq population using the iHS, xp-EHH and PBE statistics. We combined the genome-wide rank of all three statistics for each SNP to compute a Fisher score called F_SC_^21^. In subsequent analyses, we defined the highest 1% of F_SC_ as outlier SNPs and targets of positive selection (Supplementary Table S1). Some of the identified candidates genes (see Supplementary Table S2 for the full list) were previously documented as being under positive selection, among other populations, in East Asians (*SLC35F3*^22^, *COL11A2*^23^), in Orang Asli populations of the Malay Peninsula (*LRP2*, *RUNX1*,* SYN3*,* STK39*)^24,25^ and specifically in Bateq and Mendriq of the Malay Peninsula (*FAM78B*, *TADA1*, *POGK*, *DUSP27*, *CNTNAP2*, *CDH11*, *RBFOX3*)^25^. Another group of the positively selected genes (*PTPRN2*, *NRXN3*, *AMZ1*, *FRAS1*) have been linked to the ‘negrito-like’ phenotypes observed in some Southeast Asians, characterized by short stature, dark skin and hair color, and tightly curled hair^26^. Over-representation analysis (ORA), using WebGestalt^27^ to perform pathway analyses using Reactome^28^ and KEGG (Kyoto Encyclopedia of Genes and Genomes)^29^ databases, revealed that the positively selected genes are enriched in biological pathways related to human diseases such as asthma or type I diabetes mellitus. The other enriched pathways range from cell adhesion molecules, over muscle contraction to extracellular matrix organization (Table 1 and Supplementary Table S3).

**Table 1:** Gene sets under positive selection in the Maniq enriched in Reactome or KEGG pathways.

Pathway database	Gene set	Description	*P*-Value	False discovery rate (FDR)
KEGG	hsa05310	Asthma	0.000009	0.002177
KEGG	hsa04940	Type I diabetes mellitus	0.000001	0.000331
KEGG	hsa04750	Inflammatory mediator regulation of TRP channels	0.000001	0.000331
KEGG	hsa04514	Cell adhesion molecules	< 0.000001	0.000063
KEGG	hsa04921	Oxytocin signaling pathway	< 0.000001	0.000151
KEGG	hsa04261	Adrenergic signaling in cardiomyocytes	0.000002	0.000475
Reactome	R-HSA-397014	Muscle contraction	< 0.000001	0.000056
KEGG	hsa04015	Rap1 signaling pathway	0.000081	0.009808
Reactome	R-HSA-1474244	Extracellular matrix organization	0.000072	0.009445
Reactome	R-HSA-112316	Neuronal System	0.000014	0.002581

**Fig. 1:**
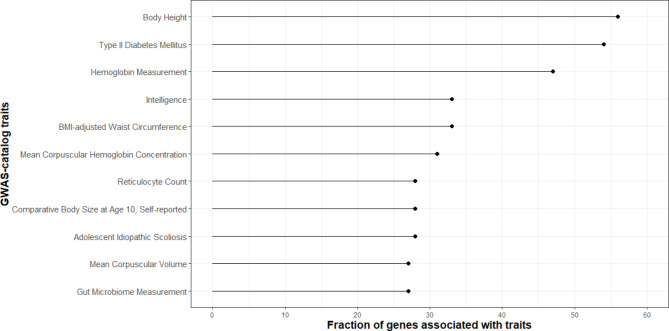
Positive selection on genes that overlap with different GWAS traits.

Figure 1 shows the highest fraction of putative positive selected genes that overlap with different GWAS traits, including body height, type II diabetes mellitus, different blood protein parameters, BMI-adjusted waist circumference, a specific type of scoliosis, and gut microbiome composition (Supplementary Table S4).

### Genome-wide signature of balancing selection

A signature of balancing selection was detected in 41 genes (Supplementary Table S5). Notably, the HLA region stands out with the highest *β(1)* values such as the genes *HLA-C* (rs1130838), *HLA-DQB1* (rs1049056), *HLA-DPA1* (rs1126769), and *HLA-DPB1* (rs1126719). The Reactome and KEGG pathway analysis revealed that only immunity-related or immunological-related biological pathways were enriched (Table 2 and Supplementary Table S6).

**Table 2:** Gene sets under balancing selection in the Maniq enriched in Reactome or KEGG pathways.

Pathway database	Gene set	Description	*P*-value	False discovery rate (FDR)
Reactome	R-HSA-202430	Translocation of ZAP-70 to Immunological synapse	0.000014	0.005565
Reactome	R-HSA-202427	Phosphorylation of CD3 and TCR zeta chains	0.000023	0.006733
Reactome	R-HSA-389948	PD-1 signaling	0.000026	0.006733
KEGG	hsa05330	Allograft rejection	0.000002	0.00238
KEGG	hsa05332	Graft-versus-host disease	0.000004	0.00238
KEGG	hsa04940	Type I diabetes mellitus	0.000004	0.00238
KEGG	hsa05320	Autoimmune thyroid disease	0.000009	0.004401
KEGG	hsa05416	Viral myocarditis	0.000024	0.006733
Reactome	R-HSA-877300	Interferon gamma signaling	0.000004	0.00238

Several other genes involved in innate immunity, such as *IL1RL1*, *GNLY*, *TNFRSF10D*, *TRIM22*, *LILRB1*, *CCL4*, and *DEFB127*, show a signature of balancing selection (Table 3).

**Table 3:** Immune genes under balancing selection in the Maniq.

Chr	Gene	Gene description
2q12.1	*IL1RL1*	Interleukin 1 receptor like 1
2p11.2	*GNLY*	Granulysin
6p211.33	*HLA-C*	Major histocompatibility complex, class I, C
6p211.32	*HLA-DPB1*, *-DQB1*, -*DPA1*	Major histocompatibility complex, class II
8p21.3	*TNFRSF10D*	Tumor necrosis factor receptor superfamily member 10D
11p15.4	*TRIM22*	Tripartite Motif Containing 22
12q12	*MUC19*	Mucin 19, oligomeric
12p13.31	*CLEC4D*	C-type lectin domain family 4 member D
14q11.2	*TRAV14DV4*	T cell receptor alpha variable 14/delta variable 4
19q13.42	*LILRB1*	Leukocyte immunoglobulin like receptor B1
17q12	*CCL4*	C-C motif chemokine ligand 4
20p13	*DEFB127*	Defensin beta 127

In addition, the *MUC19*, which shows a signature of balancing selection, encodes mucin, a major component of mucus, which is part of the innate immune responses. Genes with a function similar to *OR51B2*, *OR51Q1*, and *OR1N2* that code for olfactory receptor molecules also show evidence of balancing selection.

## Discussion

Our analysis of the Maniq autosomal genome provides valuable insights into the interplay between positive and balancing selection in shaping their unique genetic makeup. By identifying signals of positive selection and balancing selection, our study elucidates the genetic underpinnings of traits that may have evolved in response to their distinct hunter-gatherer lifestyle and rainforest environment. One of the key findings is the identification of genes under positive selection that are enriched in biological pathways indicative of adaptations related to immunity and metabolism, structural and locomotor adaptations, adaptations related to cardiovascular functions, and neuromodulatory traits (Table 1, Supplementary Table S3). For instance, the enrichment of positive selected genes in the type I diabetes mellitus pathway suggests a connection between immunity and metabolic regulation, likely driven by the ecological and dietary challenges of the rainforest. The enrichment of genes in the oxytocin signaling pathway may reflect adaptations to physiological processes such as labour (uterine contractility) and lactation, but could also be related to the Maniq’s cooperative social structure, stress modulation, and parenting behaviors, which would be critical in their egalitarian and group-dependent hunter-gatherer lifestyle. The adrenergic signaling in cardiomyocytes pathway highlights potential cardiovascular adaptations, such as enhanced resilience to stress and improved physical performance, which are vital for survival in resource-scarce and physically demanding rainforest habitats. Similarly, the extracellular matrix organization pathway, enriched with collagen-related genes (*COL11A2*, *COL5A1*, *COL4A2*, *COL18A1*), highlights adaptations for structural support and mobility, potentially critical adaptations for life in the humid, dense rainforest. Notably, *COL11A2* has been repeatedly identified as a target of positive selection in other populations, including East Asians, the Orang Asli^26^, indigenous Mesoamericans^33^, and the Luhya in Webuye, Kenya^23^, suggesting its importance in fibrillogenesis and structural adaptation.

We also identified several genes, including *SLC35F3*, *LRP2*, *RUNX1*,* SYN3*, *STK39*,* FAM78B*, *TADA1*, *POGK*, *DUSP27*, *CNTNAP2*, *CDH11*, *RBFOX3*,* PTPRN2*, *NRXN3*, *AMZ1*, and *FRAS1*, which have previously been identified as undergoing positive selection in various Asian populations (Supplementary Table S2). Specifically, several of the genes mentioned above (*PTPRN2*, *NRXN3*, *AMZ1*, *FRAS1*) are under positive selection in different Orang Asli populations and have been linked to the so-called Southeast Asian ‘negrito-like’ phenotype (i.e., small body height, curled black hair color and hair shape)^26^. While these results clearly add evidence for shared genotypes among these populations, the specific genes responsible for the claimed ‘negrito-like’ phenotype remain uncertain.

Height, a highly polygenic trait with over 700 associated loci^34^, reflects a complex interplay of genetic and environmental factors. Our analysis identified, amongst others, the gene *DNM3* as being under positive selection in the Maniq population, similar to a finding about the Batak in the Philippines^7^. The short stature of tropical hunter-gatherers has been hypothesized to reflect genetic adaptation to ecological conditions including thermoregulation, reduced food energy requirements, agile tree climbing, and rapid movement in dense rainforests^8,10–12,30^. An alternative hypothesis proposes that short stature in tropical hunter-gatherers is an outcome of a life history trade-off between accelerated growth and early sexual maturation in high-mortality environment, where early sexual maturation and reproduction is favored (fertility benefits)^7,31^ (but see^32^). Nonetheless, we did not find genes or gene-networks related with reproduction under positive selection. Our results therefore support the theory that short stature among rainforest hunter-gatherers is primarily the result of genetic adaptation to similar ecological conditions experienced by these populations. Historically the height of ‘negritos’ (including Maniq, called ‘Tonga’ in this publication) was measured around 150 cm^33^. From anectodical observations of our fieldwork we would estimate of the average of the main Maniq group we were with, to be more around 160 cm, with some (young) Maniq being around 175 cm tall. We assume that this is mainly due to increased access to rice and other nutrient rich foods. While this change in diet, can lead to increased height, it could also have adverse effects. Several genes putatively under positive selection in the Maniq are associated with GWAS traits like type II diabetes mellitus, body height, or BMI-adjusted waist circumferences (Fig. 1) suggesting the possibility of polygenic selection. If polygenic selection has indeed contributed to the so-called ‘thrifty genotype’, as proposed by Neel in 1962^34^, it could confer advantages to the Maniq population by efficient food sequestration and optimal fat storage during times of food abundance. However, the transition to a sedentary lifestyle in an agricultural or industrialized setting may lead to health issues associated with certain GWAS traits, such as obesity and its consequences, as observed in other populations^35^.

The Maniq inhabit a tropical, humid environment in the rainforests of southern Thailand. Pathogen pressure is therefore expected to be an important selective force acting on HLA genes as well as on genes of the innate immune system. Reactome analysis identified the immunity pathway ‘phosphorylation of CD3 and TCR zeta chains’ to be enriched with genes under positive selection as well as balancing selection (Table 2, Supplementary Tables S3 and S6). Among these genes are the HLA genes, which encode molecules crucial for presenting antigens on cell surfaces to T cells. HLA molecules play a pivotal role in mediating the adaptive immune response, and numerous studies have linked HLA diversity to susceptibility to major infectious diseases^36^. It has been suggested that HLA diversity is mainly influenced by pathogen-driven selection^37^. The *PTPRC* gene, encoding the CD45 molecule essential for antigen recognition by T and B lymphocytes in viral-host interactions^38^, was also found to be under positive selection in the present study, suggesting potential adaptation to pathogen pressure in the tropical rainforest environment inhabited by the Maniq population. This gene was also under positive selection in indigenous individuals living in the low Amazon tropical forest^39^, reinforce the role of pathogen pressure in shaping immune system diversity in rainforest-dwelling groups.

We also found evidence for balancing selection, particularly among several immune-related genes. The HLA genes show the highest *β(1)* scores, confirming other studies identifying the HLA genomic region as a prime region for balancing selection^40^. Several of the genes involved in immune response under balancing selection in the Maniq were already found to be under balancing selection in previous studies. These include *IL1RL1*^41^, *TRIM22*^42,43^ and *DEFB127*^44^. Balancing selection was also found in the *MUC19* gene, which may play a role in mucosal immunity, such as against human metapneumovirus infection^45^. This gene is also under positive selection in North American indigenous populations^46^, underscoring its potential importance in immune defence among diverse populations. The high prevalence of Streptococcus, Neisseria, and Haemophilus in the upper respiratory tract of the Orang Asli^47^ further supports the role of *MUC19* in bacterial clearance, given its expression in the salivary glands, where it contributes to saliva’s gel-forming mucin^48^.

Lastly, we found signatures of balancing selection for genes encoding olfactory receptor molecules. Prior research has suggested that the diversity of certain human olfactory receptors may be maintained by balancing selection, possibly through mechanisms such as overdominance^49^. In the latter study the gene *OR1N2*, which is under balancing selection in the Maniq, was also under balancing selection in the Yoruba population in Ibadan, Nigeria (1000 Genomes population). These findings support the hypothesis that genetic diversity in certain human olfactory receptor genes has been shaped by balancing selection, potentially over extended periods through mechanisms such as overdominance^50^.

In our previous study^2^, we provide evidence that the Maniq always have been a small population with a long history of isolation and endogamy. Current estimates on the population size of the Maniq range from 300 to 350 people, estimations before 1960 ranged from 100 to 300 people^33^. The extremely high genetic drift of the Maniq could, together with the small sample size, influence our results, although it is unclear in which direction. However, the detection of positive selection in this study was strengthened by employing multiple complementary approaches, including iHS, xp-EHH, and PBE statistics. By integrating these methods and calculating a Fisher score, we ensured a robust identification of genomic regions under positive selection. As with many genetic studies, a higher sample size as well as data from full genome sequencing, would allow for more comprehensive conclusions.

In summary, our study provides novel insights into the genetic adaptations of the Maniq population, highlighting the interplay of positive and balancing selection in shaping traits critical for survival in their rainforest environment. Genes under positive selection were enriched in pathways related to immunity, metabolism, structural integrity, cardiovascular performance, and neuromodulatory signaling, reflecting adaptations to environmental challenges, pathogen pressure, and the demands of a hunter-gatherer lifestyle. Evidence of balancing selection in immune-related genes highlights the critical role of maintaining genetic diversity in a pathogen-rich environment, ensuring robust defenses against a wide array of infectious threats. These findings enhance our understanding of the evolutionary pressures faced by rainforest hunter-gatherers.

## Materials and methods

### Genomic data preparation and phasing

This study is based on SNP genotype data of 21 Maniq individuals obtained in our previous study using the Infinium^®^ Omni2.5-8 v1.3 BeadChip (Illumina Inc., San Diego, CA). This SNP chip is enriched with an additional 200k exonic SNPs, yielding over 2.5 million markers from autosomes, sex chromosomes, and mitochondrial DNA. The SNP data were processed using PLINK 1.9 software for quality checking and filtering^51^. Mitochondrial and sex chromosomal SNPs were removed along with variants that are non-biallelic or duplicates. We included only SNPs with a genotyping rate of 90% (*--geno* 0.1), and all SNPs that deviated from Hardy-Weinberg equilibrium were filtered out using the *--hwe midp* threshold filter (with a *p*-value < 1e−6). The Michigan Imputation Server (MIS) (https://imputationserver.sph.umich.edu/index.html) was used for phasing and imputation of the autosomal genotype data applying the Minimac4 algorithm^52^. We followed the recommended MIS data preparation guidelines (https://imputationserver.readthedocs.io/en/latest/prepare-your-data/) for pre-imputation checks using the imputation preparation tools available at https://www.well.ox.ac.uk/~wrayner/tools/ (the Perl script HRC-1000G-check-bim-v4.2.13). As reference panel we used the ASIA genome reference legend (available at https://www.well.ox.ac.uk/~wrayner/tools/ASIA.Genome.Reference.legend.zip); we specified as population Southeast Asia (*SEA*) using the provided Perl script *HRC-1000G-check-bim.pl -b < bim file> -f < Frequency file> -r ASIA.Genome.Reference.legend -g -p SEA*; we did not apply any SNP frequency threshold. The Asian reference panel derived from the GenomeAsia 100 K Project^53^, which also includes genome sequences of the Semang (Malay Negrito) and Onge (Andamanese). The provided VcfCooker software (https://genome.sph.umich.edu/wiki/VcfCooker) was used to convert PLINK (binary) format to VCF format. The autosomal SNP genotype files were aligned to the human reference sequence (GRCh37/hg19: http://ftp.1000genomes.ebi.ac.uk/vol1/ftp/technical/reference/). For post-imputation control, MIS provides an imputation quality (*MACH-Rsq*) metrics file (.*info* files). This also includes *Rsq* as a quality metric presenting the estimated value of the squared correlation between imputed genotypes and true, unobserved genotypes. We excluded all SNPs with a *Rsq* threshold < 0.8. The final imputed dataset, after filtering, contained 3,098,600 autosomal SNPs. In addition, we included in this study autosomal SNP genotype data from the 1000 Genomes project (phase 3; ftp://ftp.1000genomes.ebi.ac.uk/vol1/ftp/release/20130502/)^54^. These data had already been prepared in a previous study^55^. We selected 25 individuals representing East Asian ancestry (EAS), South Asian ancestry (SAS), European ancestry (EUR), and African ancestry (AFR) from the following populations: CHB (Han Chinese in Beijing, China), BEB (Bengali from Bangladesh), GBR (British in England and Scotland), and LWK (Luhya in Webuye, Kenya). The data were finally merged with the Maniq genotype data using PLINK (--bmerge).

### Identifying signatures of positive directional selection

To detect positive selection (selective sweeps) in the phased autosomal chromosomes, we employed the integrated haplotype score (iHS) approach^16^, the cross-population extended haplotype homozygosity (xp-EHH)^17^ approach, and the Population Branch Excess (PBE) statistics^18^ and combined all three methods by calculating a Fisher score. The iHS method captures the ratio of extended haplotype homozygosity (EHH) for the haplotypes carrying the derived iHHD and ancestral allele iHHA at candidate SNP sites. The required genetic maps were obtained from HapMap phase II b37^56^. We used the software selscan, version 1.2.0a (https://github.com/szpiech/selscan)^57^, to calculate iHS scores for non-overlapping windows of 100 kilobase pairs (kb); iHS scores for core sites with a minor allele frequency (MAF) < 0.05 are not calculated by the program; the unstandardized iHS scores were normalized in default frequency bins (--bins 100) across the entire genome using the script norm provided by the selscan programme.

The xp-EHH algorithm compares the extended haplotype homozygosity (EHH) at a given locus in one population to that in a reference population to detect differences in the decay of haplotype homozygosity between the two populations. A positive xp-EHH score indicates longer haplotype homozygosity in the test population, which suggests that positive selection has acted more strongly on the focal population relative to the reference population. Conversely, a negative xp-EHH score implies stronger positive selection in the reference population. We computed xp-EHH scores between the Maniq population and as reference population, the 1000 Genomes East Asian population, Kinhin Ho Chi Minh City (KHV), from Vietnam, using selscan software, version 1.2.0a^57^. The xp-EHH scores were calculated for non-overlapping windows of 100 kilobase pairs (kb) across the autosomal chromosomes. Unstandardized xp-EHH values were normalized using default parameters (--bins 100) to account for allele frequency differences between populations. Pairwise *F*_*ST*_ (Maniq and 1000 Genomes populations) were calculated using Weir & Cockerham *F*_*ST*_ calculation implemented in VCFtools^58,59^. Negative *F*_*ST*_ values were set to zero. The PBE method^18^, is a modified approach of the original Population Branch Statistics (PBS)^60^. PBS is calculated using *F*_*ST*_ values between three populations: the focal population (A), and two reference populations (B and C). The PBE approach considers the excess branch length that is specific to the focal population by incorporating additional genomic context and population structure information. We implemented the *PBE* calculation using custom scripts in R (version 4.1.0)^61^ using the formula as described by Yassin in 2016^18^:

$$PBE=PB{S_{obs}} - PB{S_{exp}}=PBS - \left[ {{T^{BC}} \times \left( {PB{S_{med}}/T_{{med}}^{{BC}}} \right)} \right].$$

PBS values are calculated using *F*_*ST*_ values between three populations: the focal population A = Maniq population, and two reference populations B = CHB (East Asian ancestry) and C = LWK (African ancestry) from the 1000 Genomes database (see above). The PBS for the focal population A is given by: *PBS*_*A*_*=* (*T*_*AB*_*+ T*_*AC*_*+ T*_*BC*_)/2 where *T*_*AB*_​, *T*_*AC*_​, and *T*_*BC*_ are the branch lengths derived from the *F*_*ST*_ values between focal and reference populations. PBE is calculated using the following formula: *PBE* = *PBS*_*obs*_−PBS_exp_. Here, *PBS*_*obs*_ represents the observed *PBS* for the focal population, and *PBS*_*exp*_ is the expected *PBS*, calculated as given in the formulae above whereby presents *T*^*BC*^ the branch length between the two reference populations B and C and serves to scale locus-specific expectations for expectations for genetic differentiation and should therefore be centred around zero (as observed by the original study^18^ and in our data—see Supplementary Figure S1); *PBS*_*med*_ is the median PBS value across the genome; $T_{{med}}^{{BC}}$ is the median branch length between the reference populations B and C across the genome. The above formula adjusts thus the PBS by subtracting the expected value, which is derived from the *PBS*_*med*_ and branch length values, thus highlighting regions with excess differentiation specific to the focal population.

We calculated the genome-wide rank for each of the three statistics independently, using ‘rank(-test_statistic, ties.method = “average”)’ in R. The absolute values of iHS, and only the positive values of xp-EHH and PBE were used to assign the respective ranks. The Fisher score F_SC_ was computed for each SNP by summing -log10(rank of the statistic/number of SNPs) over all three tests^21^. In this study, we considered SNPs as outliers and candidates under positive selection if they were among the highest 1%. The Ensembl Variant Effect Predictor programme package (https://github.com/Ensembl/ensembl-vep)^62^ was used to map genetic information such as gene symbol and consequence type to the SNP data.

### Identifying signatures of balancing selection

We used BetaScan v1.0 software (https://github.com/ksiewert/BetaScan) to detect the signature of long-term balancing selection^19^. In addition, the toolkit of the glactools programme (https://grenaud.github.io/glactools/)^63^ was used to convert the phased chromosomes to folded site frequency spectra format in accordance with the requirements of the BetaScan software (*glactools vcfm2acf vcfm2acf --onlyGT --fai human_g1k_v37.fasta.fai*) to *acf* format. We used as window size 1kbp around the core SNP. This software calculates the allele frequency correlation summary statistic *β(1)* to detect balancing selection using polymorphism data. A characteristic of balancing selection is high polymorphism and an excess of alleles at intermediate frequencies. The *ß* statistic scans for an excess of genomically proximate SNPs that have a very similar allele frequency surrounding a core balanced SNP and then weights the sum of SNP counts around the core SNP higher if they have very similar frequencies to the core SNP. These SNP clusters of SNPs at highly similar intermediate frequency are consistent with the action of balancing selection and are summarized as the *β(1)* statistic, where high values indicate balancing selection. Balancing selection is expected to decrease *F*_*ST*_ among populations^64^. We calculated in R genome-wide empirical *p*-values using 100 permutations. Because of the size of the data and number of permutations, we ran this test separately for each chromosome and then calculated the average significance value across all autosomes. We thus report *β(1)* ≥ 6.3 as statistically significant values (*p* < 0.01) and report only functional SNP polymorphism, i.e., missense, synonymous variants, and SNPs that are located in the 3’ UTR or 5’ UTR.

### Pathway and gene genontology (GO) analysis

The web-based gene set analysis toolkit (WebGestalt)^27^ was used for an overrepresentation analysis (ORA) to test whether genes under selection are enriched in certain biological pathways. In our WebGestalt analyses we included pathways from the functional databases Reactome^28^ and KEGG^29^. WebGestalt employs the Benjamini-Hochberg (BH) procedure to take multiple testing into account with a false discovery rate (FDR) < 0.01. If more than 10 pathways were found to be enriched, we used the provided weighted set cover method to reduce redundancy in results. We accessed genome-wide association data (GWAS) from the NHGRI-EBI GWAS catalog (https://www.ebi.ac.uk/gwas/)^65^ (accessed between October and November 2024) to obtain data on human traits associated with genes under selection. In addition, we used the web-server application snpXplorer (https://snpxplorer.net/)^66^ to explore association statistics from published GWAS. The approach examines the proportion of genes associated with the input SNPs for which a previous association was reported in the GWAS catalog. Further information on genes, like gene type or description was obtained using BioMart of Ensembl^67^ (https://www.ensembl.org/biomart/martview/).

## Electronic supplementary material

Below is the link to the electronic supplementary material.

Supplementary Material 1

Supplementary Material 2

Supplementary Material 3

Supplementary Material 4

Supplementary Material 5

Supplementary Material 6

Supplementary Material 7

## Data Availability

The genomic data can be obtained from 1000 Genomes database. The data from the Maniq generated in this study are not openly available due to reasons of sensitivity and will be made available to researchers upon reasonable request after review and approval by the Data Access Committee led by the corresponding authors.

## References

[1] Fix, A. G. Malayan paleosociology—implications for patterns of genetic-variation among the orang-asli. *Am. Anthropol.* **97**, 313–323 (1995).

[2] Goellner, T. et al. Unveiling the genetic history of the Maniq, a primary Hunter-Gatherer Society. *Genome Biol. Evol.* **14**, evac021 (2022). 10.1093/gbe/evac021 PMC9005329 35143674

[3] Lukas, H. Can they save us, the foragers? Indonesian and Thai Hunter-Gatherer cultures under threat from outside (2004).

[4] Kricheff, D. A. & Lukas, H. Being Maniq: practice and identity in the forests of Southern Thailand. *Hunt. Gatherer Res.* **1**, 139–155 (2015).

[5] Khunweechuay, N., Roongtawanreongsri, S. & Hatta, K. Cultural forest ecosystem services of the Maniq Indigenous People in Southern Thailand. *Hum. Ecol.* **50**, 559–576 (2022).

[6] Rees, J. S., Castellano, S. & Andrés, A. M. The genomics of human local adaptation. *Trends Genet.* **36**, 415–428 (2020). 32396835 10.1016/j.tig.2020.03.006

[7] Migliano, A. B. et al. Evolution of the pygmy phenotype: evidence of positive selection from genome-wide scans in African, Asian, and melanesian pygmies. *Hum. Biol.* **85**, 251–284 (2013). 24297229 10.3378/027.085.0313

[8] Hsieh, P. et al. Whole-genome sequence analyses of western Central African pygmy hunter-gatherers reveal a complex demographic history and identify candidate genes under positive natural selection. *Genome Res.* **26**, 279–290 (2016). 26888263 10.1101/gr.192971.115 PMC4772011

[9] Perry, G. H. & Verdu, P. Genomic perspectives on the history and evolutionary ecology of tropical rainforest occupation by humans. *Quat Int.* **448**, 150–157 (2017).

[10] Bergey, C. M. et al. Polygenic adaptation and convergent evolution on growth and cardiac genetic pathways in African and Asian rainforest hunter-gatherers. *Proc. Natl. Acad. Sci. U. S. A.* **115**, E11256–E11263 (2018). 10.1073/pnas.1812135115 PMC6275523 30413626

[11] Little, M. A. Evolutionary strategies for body size. *Front. Endocrinol.* **11**, 107 (2020). 10.3389/fendo.2020.00107 PMC7075806 32210916

[12] Lopez, M. et al. Genomic evidence for local adaptation of hunter-gatherers to the African rainforest. *Curr. Biol.* **29**, 2926–2935 (2019). 31402299 10.1016/j.cub.2019.07.013

[13] Scheinfeldt, L. B. et al. Genomic evidence for shared common ancestry of East African hunting-gathering populations and insights into local adaptation. *Proc. Natl. Acad. Sci. U. S. A.* **116**, 4166–4175 (2019). 10.1073/pnas.1817678116 PMC6410815 30782801

[14] Hedrick, P. W. Balancing selection. *Curr. Biol.* **17**, R230–R231 (2007). 17407748 10.1016/j.cub.2007.01.012

[15] Bitarello, B. D., Brandt, D. Y. C., Meyer, D. & Andrés, A. M. Inferring balancing selection from genome-scale data. *Genome Biol. Evol.* **15**, 785 (2023). 10.1093/gbe/evad032 PMC10063222 36821771

[16] Voight, B. F., Kudaravalli, S., Wen, X. Q. & Pritchard, J. K. A map of recent positive selection in the human genome. *Plos Biol.* **4**, 659–659 (2006). 10.1371/journal.pbio.0040072 PMC1382018 16494531

[17] The International HapMap Consortium. Genome-wide detection and characterization of positive selection in human populations. *Nature* **449**, 913–918 (2007). 17943131 10.1038/nature06250 PMC2687721

[18] Yassin, A. et al. Recurrent specialization on a toxic fruit in an island Drosophila population. *Proc. Natl. Acad. Sci. U S A*. **113**, 4771–4776 (2016). 27044093 10.1073/pnas.1522559113 PMC4855561

[19] Siewert, K. M. & Voight, B. F. Detecting long-term balancing selection using allele frequency correlation. *Mol. Biol. Evol.* **34**, 2996–3005 (2017). 28981714 10.1093/molbev/msx209 PMC5850717

[20] Siewert, K. M. & Voight, B. F. BetaScan2: standardized statistics to detect balancing selection utilizing substitution data. *Genome Biol. Evol.* **12**, 3873–3877 (2020). 32011695 10.1093/gbe/evaa013 PMC7058154

[21] Patin, E. et al. Dispersals and genetic adaptation of Bantu-speaking populations in Africa and North America. *Science* **356**, 543–546 (2017). 28473590 10.1126/science.aal1988

[22] Ma, X. X. & Xu, S. H. Archaic introgression contributed to the pre-agriculture adaptation of vitamin B1 metabolism in East Asia. *Iscience* **25**, 12 (2022). 10.1016/j.isci.2022.105614 PMC9712685 36465121

[23] Liu, X. Y. et al. Detecting and characterizing genomic signatures of positive selection in global populations. *Am. J. Hum. Genet.* **92**, 866–881 (2013). 23731540 10.1016/j.ajhg.2013.04.021 PMC3675259

[24] Deng, L. et al. The population genomic landscape of human genetic structure, admixture history and local adaptation in Peninsular Malaysia. *Hum. Genet.* **133**, 1169–1185 (2014). 24916469 10.1007/s00439-014-1459-8

[25] Liu, X. Y. et al. Differential positive selection of malaria resistance genes in three indigenous populations of Peninsular Malaysia (134, pg 375, 2015). *Hum. Genet.* **134**, 677–677 (2015). 25634076 10.1007/s00439-014-1525-2

[26] Deng, L. A. et al. Genetic connections and convergent evolution of tropical indigenous peoples in Asia. *Mol. Biol. Evol.* **39**, 456 (2022). 10.1093/molbev/msab361 PMC8826522 34940850

[27] Liao, Y. X., Wang, J., Jaehnig, E. J., Shi, Z. A. & Zhang, B. WebGestalt 2019: gene set analysis toolkit with revamped UIs and APIs. *Nucleic Acids Res.* **47**, W199–W205 (2019). 31114916 10.1093/nar/gkz401 PMC6602449

[28] Milacic, M. et al. The reactome pathway knowledgebase 2024. *Nucleic Acids Res.*10.1093/nar/gkad1025 (2023). 10.1093/nar/gkad1025 PMC10767911 37941124

[29] Kanehisa, M., Furumichi, M., Sato, Y., Kawashima, M. & Ishiguro-Watanabe M. KEGG for taxonomy-based analysis of pathways and genomes. *Nucleic Acids Res.* **51**, D587–D592 (2023). 36300620 10.1093/nar/gkac963 PMC9825424

[30] H Perry, G. et al. Adaptive, convergent origins of the pygmy phenotype in African rainforest hunter-gatherers. *Proc. Natl. Acad. Sci.* **111**, E3596–E3603 (2014). 25136101 10.1073/pnas.1402875111 PMC4156716

[31] Migliano, A. B., Vinicius, L. & Lahr, M. M. Life history trade-offs explain the evolution of human pygmies. *Proc. Natl. Acad. Sci. U S. A.* **104**, 20216–20219 (2007). 18077366 10.1073/pnas.0708024105 PMC2154411

[32] S. A. Becker, N., Verdu, P., Hewlett, B. & Pavard, S. Can life history trade-offs explain the evolution of short stature in human pygmies? A response to Migliano et al. (2007). *Hum. Biol.* **82**, 17–27 (2010). 20504169 10.3378/027.082.0101

[33] Brandt, J. H. The negrito of peninsular Thailand. *J. Siam Soc.* **49**, 123–158 (1961).

[34] Neel, J. V. Diabetes mellitus—a thrifty genotype rendered detrimental by progress. *Am. J. Hum. Genet.* **14**, 353 (1962). 13937884 PMC1932342

[35] Pressler, M. et al. Dietary transitions and health outcomes in four populations—systematic review. *Front. Nutr.* **9**, 748305 (2022). 10.3389/fnut.2022.748305 PMC8892920 35252289

[36] Alicia, S. M. A review of HLA allele and SNP associations with highly prevalent infectious diseases in human populations. *Swiss Med. Wkly.* **150**, 20214 (2020). 10.4414/smw.2020.20214 32297957

[37] Prugnolle, F. et al. Pathogen-driven selection and worldwide HLA class I diversity. *Curr. Biol.* **15**, 1022–1027 (2005). 15936272 10.1016/j.cub.2005.04.050

[38] Saunders, A. E. & Johnson, P. Modulation of immune cell signalling by the leukocyte common tyrosine phosphatase, CD45. *Cell. Signal.* **22**, 339–348 (2010). 19861160 10.1016/j.cellsig.2009.10.003

[39] Borda, V. et al. The genetic structure and adaptation of Andean highlanders and amazonians are influenced by the interplay between geography and culture. *Proc. Natl. Acad. Sci. U. S. A.* **117**, 32557–32565 (2020). 33277433 10.1073/pnas.2013773117 PMC7768732

[40] Barreiro, L. B. & Quintana-Murci, L. From evolutionary genetics to human immunology: how selection shapes host defence genes. *Nat. Rev. Genet.* **11**, 17–30 (2010). 19953080 10.1038/nrg2698

[41] Meyer, D., Aguiar, V. R. C., Bitarello, B. D., Brandt, D. Y. C. & Nunes, K. A genomic perspective on HLA evolution. *Immunogenetics* **70**, 5–27 (2018). 28687858 10.1007/s00251-017-1017-3 PMC5748415

[42] Andrés, A. M. et al. Targets of balancing selection in the human genome. *Mol. Biol. Evol.* **26**, 2755–2764 (2009). 19713326 10.1093/molbev/msp190 PMC2782326

[43] Kelly, J. N., Woods, M. W., Xhiku, S. & Barr, S. D. Ancient and recent adaptive evolution in the antiviral TRIM22 gene: identification of a single-nucleotide polymorphism that impacts TRIM22 function. *Hum. Mutat.* **35**, 1072–1081 (2014). 24863734 10.1002/humu.22595

[44] Hollox, E. J. & Armour, J. A. L. Directional and balancing selection in human beta-defensins. *Bmc Evol. Biol.* **8**, 1–14 (2008). 10.1186/1471-2148-8-113 PMC2373304 18416833

[45] McBride, K., Banos-Lara, M. D., Cheemarla, N. R. & Guerrero-Plata, A. Human metapneumovirus induces mucin 19 which contributes to viral pathogenesis. *Pathogens* **9**, 726 (2020). 10.3390/pathogens9090726 PMC7559929 32899224

[46] Reynolds, A. W. et al. Comparing signals of natural selection between three indigenous north American populations. *Proc. Natl. Acad. Sci. U S A* **116**, 9312–9317 (2019). 30988184 10.1073/pnas.1819467116 PMC6511053

[47] Cleary, D. W. et al. The upper respiratory tract microbiome of indigenous Orang Asli in north-eastern Peninsular Malaysia. *Npj Biofilms Microbiomes* **7**, 1 (2021). 10.1038/s41522-020-00173-5 PMC7785749 33402693

[48] Culp, D. J. et al. Salivary mucin 19 glycoproteins innate immune functions in streptococcus mutans-induced caries in mice and evidence for expression in human saliva. *J. Biol. Chem.* **290**, 2993–3008 (2015). 25512380 10.1074/jbc.M114.597906 PMC4317041

[49] Alonso, S., López, S., Izagirre, N. & de la Rua, C. Overdominance in the human genome and olfactory receptor activity. *Mol. Biol. Evol.* **25**, 997–1001 (2008). 18296703 10.1093/molbev/msn049

[50] Olender, T. et al. Personal receptor repertoires: olfaction as a model. *Bmc Genom* **13**, 1–16 (2012). 10.1186/1471-2164-13-414 PMC3462693 22908908

[51] Chang, C. C. et al. Second-generation PLINK: rising to the challenge of larger and richer datasets. *Gigascience* **4**, 13742 (2015). 10.1186/s13742-015-0047-8 PMC4342193 25722852

[52] Das, S. et al. Next-generation genotype imputation service and methods. *Nat. Genet.* **48**, 1284–1287 (2016). 27571263 10.1038/ng.3656 PMC5157836

[53] Wall, J. D. et al. The GenomeAsia 100K Project enables genetic discoveries across Asia. *Nature* **576**, 106 (2019). 31802016 10.1038/s41586-019-1793-z PMC7054211

[54] Auton, A. et al. A global reference for human genetic variation. *Nature* **526**, 68–74 (2015). 26432245 10.1038/nature15393 PMC4750478

[55] Schaschl, H., Göllner, T. & Morris, D. L. Positive selection acts on regulatory genetic variants in populations of European ancestry that affect ALDH2 gene expression. *Sci. Rep.* **12**, 4563 (2022). 10.1038/s41598-022-08588-0 PMC8927298 35296751

[56] Altshuler, D. M. et al. Integrating common and rare genetic variation in diverse human populations. *Nature* **467**, 52–58 (2010). 20811451 10.1038/nature09298 PMC3173859

[57] Szpiech, Z. A. & Hernandez, R. D. Selscan: an efficient multithreaded program to perform EHH-based scans for positive selection. *Mol. Biol. Evol.* **31**, 2824–2827 (2014). 25015648 10.1093/molbev/msu211 PMC4166924

[58] Weir, B. S. & Cockerham, C. C. Estimating F-statistics for the analysis of population-structure. *Evolution* **38**, 1358–1370 (1984). 28563791 10.1111/j.1558-5646.1984.tb05657.x

[59] Danecek, P. et al. The variant call format and VCFtools. *Bioinformatics* **27**, 2156–2158 (2011). 21653522 10.1093/bioinformatics/btr330 PMC3137218

[60] Yi, X. et al. Sequencing of 50 human exomes reveals adaptation to high altitude. *Science* **329**, 75–78 (2010). 20595611 10.1126/science.1190371 PMC3711608

[61] Team, R. C. R: A language and environment for statistical computing. R Foundation for Statistical Computing, Vienna, Austria. https://www.R-project.org/ (2024).

[62] McLaren, W. et al. The ensembl variant effect predictor. *Genome Biol.* **17**, 1–14 (2016). 10.1186/s13059-016-0974-4 PMC4893825 27268795

[63] Renaud, G. Glactools: a command-line toolset for the management of genotype likelihoods and allele counts. *Bioinformatics* **34**, 1398–1400 (2018). 29186325 10.1093/bioinformatics/btx749

[64] Nielsen, R. Molecular signatures of natural selection. *Annu. Rev. Genet.* **39**, 197–218 (2005). 16285858 10.1146/annurev.genet.39.073003.112420

[65] Buniello, A. et al. The NHGRI-EBI GWAS catalog of published genome-wide association studies, targeted arrays and summary statistics 2019. *Nucleic Acids Res.* **47**, D1005–D1012 (2019). 30445434 10.1093/nar/gky1120 PMC6323933

[66] Tesi, N., van der Lee, S., Hulsman, M., Holstege, H. & Reinders, M. J. T. snpXplorer: a web application to explore human SNP-associations and annotate SNP-sets. *Nucleic Acids Res.* **49**, W603–W612 (2021). 34048563 10.1093/nar/gkab410 PMC8262737

[67] Harrison, P. W. et al. Ensembl 2024. *Nucleic Acids Res.* **52**, D891–D899 (2024). 37953337 10.1093/nar/gkad1049 PMC10767893

