# Supplementary material for: Natural selection and adaptive traits in the Maniq, a nomadic hunter-gatherer society from Mainland Southeast Asia
Source: Sci Rep. 2025 Feb 9;15:4809. doi: 10.1038/s41598-024-83657-0 (PMC11808089; doi:10.1038/s41598-024-83657-0)

**Supplementary Figure S1.** Calculated distribution of PBE values of the Maniq population with 99.5^th^ percentile cutoff. The expectation is that PBE centered around zero; it will be positive to the degree that PBS exceeds its predicted value (population A), and it will be negative if there is elevated genetic differentiation specific to population B or C.


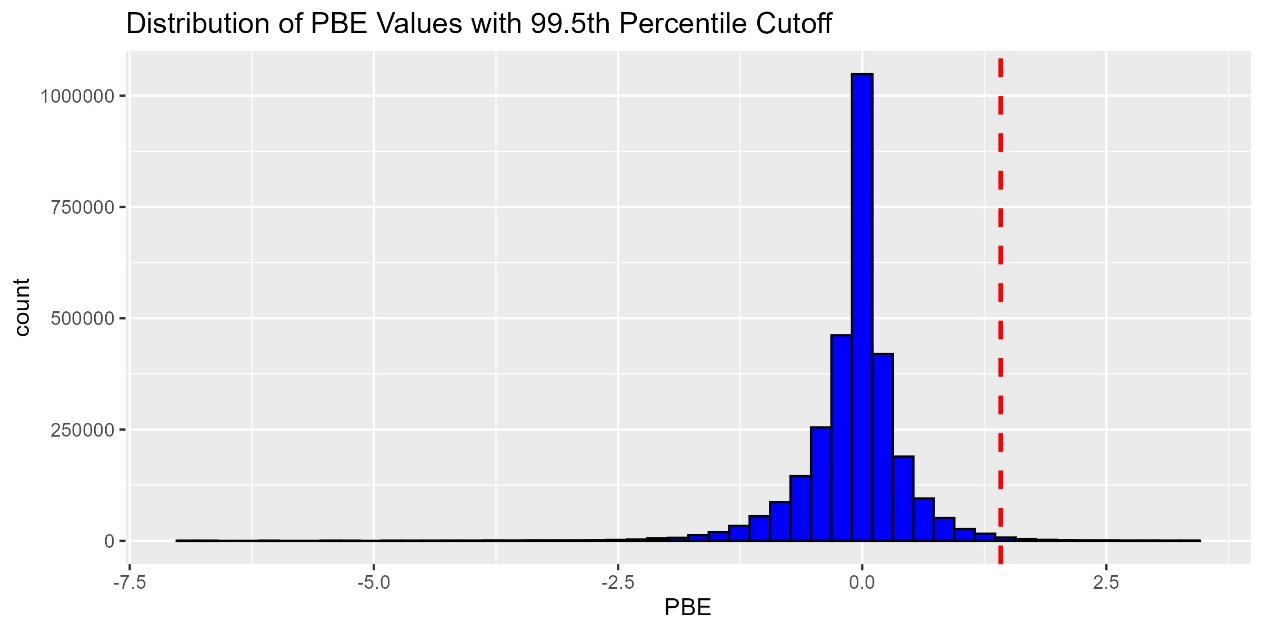

Supplement: Supplementary file 1 — Supplementary Material 1 [file 41598_2024_83657_MOESM1_ESM.docx]
